# Motivation to Care: A Qualitative Study on Iranian Nurses

**DOI:** 10.1097/jnr.0000000000000294

**Published:** 2019-07-16

**Authors:** Neda ASADI, Robabeh MEMARIAN, Zohreh VANAKI

**Affiliations:** 1 RN, Doctoral Student, Faculty of Medical Sciences, Department of Nursing, Tarbiat Modares University, Tehran, Iran; 2 PhD, RN, Assistant Professor, Faculty of Medical Sciences, Department of Nursing, Tarbiat Modares University, Tehran, Iran; 3 PhD, RN, Associate Professor, Faculty of Medical Sciences, Department of Nursing, Tarbiat Modares University, Tehran, Iran.

**Keywords:** nursing, motivation, quality care

## Abstract

**Background:**

The performance of nurses, which is rooted in personal motivation, determines the quality of care. Therefore, it is important that nurses are motivated to provide high-quality care.

**Purpose:**

The purpose of this study was to explore the factors that affect caring motivation from the perspectives of nurses in Iran.

**Methods:**

This was a qualitative study. Seventeen nurses were interviewed using a semistructured, in-depth interview method. The interviews were recorded, transcribed verbatim, and analyzed using content analysis.

**Results:**

Data analysis led to the identification of the two themes of (a) self-valuation and (b) providing beneficial care.

**Conclusions/Implications for Practice:**

The findings of this study increase scholarly understanding of the caring motivations of nurses. These motivations may be used in related programs by senior nursing managers to improve the quality of nursing care.

## Introduction

Motivation helps persuade and guide people to perform a special activity (Tahrekhani & Sadeghian, 2015) and has been considered as a prerequisite for better organizational performance (Aduo-Adjei, Emmanuel, & Forster, 2016). Motivation has an essential role to play in addressing challenges in the health department (Aduo-Adjei et al., 2016), underscoring that achieving health goals is greatly dependent on motivated healthcare specialists providing effective, efficient, and high-quality services (Aduo-Adjei et al., 2016). Nurses are responsible for providing a large part of healthcare services (Jooste & Hamani, 2017). Their performance is rooted in personal motivation (Toode, Routasalo, & Suominen, 2011) and determines to a great extent the quality of patient care (Jooste & Hamani, 2017).

Because motivation is not an observable phenomenon, studies have focused on examining its effective factors and outcomes. Studies have proven the importance of motivation in nurse retention and job satisfaction and associated motivation with these factors (Bonenberger, Aikins, Akweongo, & Wyss, 2014). In a nationwide study, Iranian nurses reported having a median level of job satisfaction (Negarandeh, Dehghan-Nayeri, & Ghasemi, 2015), with only one third of the nurses reporting being satisfied with their jobs (Valizadeh et al., 2016). This matter suggests a potential threat to the quality of patient care, as one of the greatest challenges to the Iranian health system is the low care quality for patients, which has been strongly associated with the self-perceived motivation of nurses (Negarandeh et al., 2015).

Trying to fulfill the expectations of nurses and to maximize their performance is a challenging task for hospital managers (Oshvandi et al., 2008). Identifying nurses' motivators may help nurse managers better identify or develop strategies to motivate nursing staffs (Negarandeh et al., 2015). Most of the recent studies in this field have adopted quantitative approaches (Koch, Proynova, Paech, & Wetter, 2014; Ojakaa, Olango, & Jarvis, 2014), and a small number of qualitative studies have identified factors that affect caring motivation in nurses (Alhyas, Nielsen, Dawoud, & Majeed, 2013).

Generally, recent studies on factors that affect motivation in nurses have been few in number and inadequate in scope (Koch et al., 2014). Personal values (Koch et al., 2014) and the specific factors influencing the caring motivation of nurses (Moody & Pesut, 2006) remain unclear. However, the process of developing motivational strategies may be made easier by determining empirical evidence and applying information on motivation to care and on the forces working to enhance or suppress caring motivation (Toode et al., 2011).

Carter and Kulbok posited that qualitative studies are more appropriate for validating the meaning and multidimensional combinations of motivation (Carter & Kulbok, 2002). Applying a qualitative approach to assessing the caring motivation of nurses is necessary when the researcher intends to inquire about the behaviors and focus on the experiences of the participants. Descriptive qualitative studies allow researchers to describe or explore a phenomenon, problem, or issue and may encompass a broad range of questions relating to lived experiences, knowledge, attitudes, feelings, perceptions, and views. Descriptive qualitative studies are less interpretive than other qualitative approaches such as phenomenological studies and grounded theory (AllahBakhshian et al., 2017).

Therefore, considering the ability of the qualitative method to discover the factors affecting caring motivation in nurses and the lack of relevant qualitative studies in this field in an Iranian cultural context, this study was designed and conducted to achieve an in-depth, comprehensive view of the factors affecting the caring motivation of Iranian nurses from the perspective of these nurses.

## Methods

This study presents the caring-motivation-related experiences of nurses in Iran. The sample consisted of 17 nurses (11 women and six men). Participants had worked as nurses for between 2 and 18 years. Two of the participants had master's degrees, and 15 had bachelor's degrees. Sampling was initially purposive, and then a theoretical sampling method was applied until data saturation was reached. The first two interviews were unstructured, and data analysis was used to extract relevant concepts. Afterward, the interviews were all semistructured. The interviews began with the general question: “Please explain a caring experience for which you were relatively more motivated.” The interviews continued with the question “How would you maintain your motivation of care? Describe your experiences.” The subsequent questions were guided based on the information provided by the participant and on the researcher's desire to clarify relevant issues.

### Ethical Considerations

Ethical approval was obtained from Tarbiat Modares University (Approval no. 52d/3711). Before participation, the goals of the study were explained to the participants and the participants provided their consent to participate and to have their interview sessions recorded. Nurses were assured that their information would remain confidential.

### Data Collection

Data were collected from June to November 2016 using semistructured interviews, which were performed and recorded in a quiet and calm room at the hospitals where the participants were working. The time and place of the interviews were determined by the participants. All of the interviews, which lasted between 30 and 60 minutes, were conducted by one researcher. The interviews were recorded and transcribed verbatim. In addition, in-field notes were taken to cover all aspects of the phenomenon.

### Data Analysis

Data were analyzed using conventional qualitative content analysis. The qualitative content analysis is a method for describing the meaning of qualitative data systematically. This method is executed by allocating consecutive parts of the categories into a standardized code format that covers all aspects and features of the targeted phenomenon in terms of both description and interpretation and is used as a flexible and systematic approach to reduce the physical size of data sets (Schreier, 2014). Transcripts were divided into the smallest constituent meaning units, after repeated reviews. Reviewing and cycling between the codes continued until codes were assigned to subcategories and categories based on semantic similarity to gradually develop a new concept of caring motivation.

To validate the data, the interviews and the five primary codes were given to the first five participants for checking. Their subsequent suggestions for modification were considered (member check). Furthermore, sampling was conducted to maximize variety (e.g., age, gender, work experience, ward) in line with the suggestion of previous studies.

## Results

Participants in this study related their experiences with regard to caring motivation. After data analysis, the two categories of “self-valuation” and “providing beneficial care” were extracted (Table 1).

**TABLE 1.**
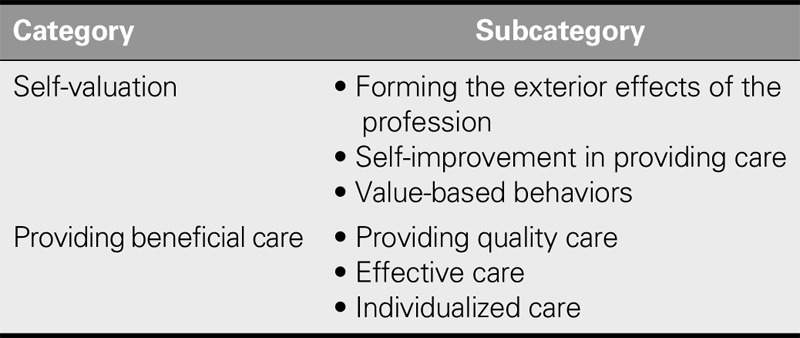
Categories and Subcategories

### Self-Valuation

Experiences and statements of the participants relevant to the “self-valuation” category were divided into three subcategories: forming the exterior effects of the profession, self-improvement in providing care, and value-based behaviors.

#### Forming the exterior effects of the profession

The participants discussed the efforts that they made to show the value of their work to others. Nurses consider patients as members of society who receive their services and who thus hold the potential to significantly affect the overall image of nursing in society. Moreover, in trying to avoid leaving a negative mental image of nursing, in addition to satisfying their patients, the participants described how they had worked to promote the exterior effects of their profession. Participant 11 (P11), a nurse from the gastroenterology ward, mentioned:

*Each patient indicates a family. We should represent our competency so that society will accept us better. We should provide science-based nursing work*.

Paying attention to interpersonal relationships was one of the dimensions of promoting the exterior effects of the profession. This matter was so important to the participants that one stated the belief that it was rooted in the dignity of their profession and, in this regard, stated:

*The dignity of our profession requires us to have ethical and professional relations with collaborative groups so that they will better recognize nurses and their abilities*. (P3)

Nurses also emphasized the importance of maintaining a face-to-face relationship with their patients. In this regard, one of the participants said:

*A nurse should not look at the serum and the device first, but should first pay attention to the patient so that the patient feels that the nurse is there for helping them, and not for checking the devices*. (P1)

Advocating the importance of nursing to others was another example of promoting the exterior effects of the profession identified in this study. In this regard, one of the participants mentioned:

*In some cases, I have talked to the doctors and persuaded them that there are better methods, and we have agreed on a patient and their treatment method. This way, they better understand my responsibilities and duties and recognize what I am doing here and the importance of what I am doing.* (P9)

#### Self-improvement in providing care

The participants reflected the presumption that self-improvement is one of the most effective methods to provide care, and they worked diligently to improve their practical and scientific competencies. The participants took responsibility for their own learning. Most expressed that, to develop their abilities, they took personal initiative to update their knowledge and that, although their organizations typically ignore retraining, they participated in workshops to gain scientific information motivated by their own personal interests:

*Most of the time I attend workshops but earn no certificate for it. But sometimes these workshops have been so useful that I wanted to go again. I am interested in learning about up-to-date information*. (P8)

In addition, participants mentioned the importance of constant learning in the field of care provision, which may affect their actual care provision practices. One of the participants said:

*Despite being tired, many times I would search new subjects. Sometimes I will search past midnight and continue studying*. (P16)

Echoing the above sentiment, P9 mentioned:

The fact is, many of the issues in our work are elusive and must constantly be reviewed. I keep summaries of articles in my locker at the hospital to review whenever I can. I have never severed my relationship with my pamphlets and books.

Furthermore, the participants related that they regularly worked to improve their communication skills. One of them noted:

*I tried to learn many things from caring, and I owe this to my profession. When I started working, through my daily communications with the patients and the colleagues, my communication techniques have changed for the better, which is natural, as long as you are willing to try.* (P12)

#### Value-based behaviors

Value-based behaviors was the third subcategory of self-valuation. Disregarding the negative aspects of the caring environment, the participants worked to separate themselves from the environment and negative environmental factors. They tried to focus their attention on their patients and to not let workplace issues affect their care provision efforts. In this regard, one of the participants said:

*I have never let my work problems interfere with my patient care. I have never thought about working less for the patients. I perform my care completely and correctly*. (P7)

Moreover, the participants expressed that they do not limit themselves to their official, expected duties and have experienced a new view toward caring to care for the patient with no expectations from others. P5 said:

*Sometimes, I would get up at 2 a.m. and perform gavage for the patient so that I would be reassured that the patient has eaten. I dedicate my break time to my patients. I do not expect anything from anybody in return.* (P5)

In addition, the participants who were in self-perceived economic need shared that they focused on their patients despite their own financial hardships. Many of the participants mentioned this matter:

*For me, low income has never made me work less than my duties require. I see no relationship between providing care and my income. If our salaries are low or delayed, it is not our patients' fault. They are innocent. The problem lies with the system.* (P9)

### Providing Beneficial Care

Experiences and statements of the participants regarding providing beneficial care were distinguished into three subcategories, which are discussed below.

#### Providing quality care

From the point of view of many participants, the most desirable manner for providing care indicated quality care. This means that care is offered safely and based on relevant nursing standards without discriminatory behaviors during the caring process. The following reflects participant experiences in this subcategory:

*When you adopt a specific format, you must abide by its standards. The standards should not be omitted. I have always tried to make the standards flexible in order to provide better-quality care*. (P4)

Most of the participants expressed the belief that quality of nursing care depended on patient satisfaction. In this regard, one of the participants stated:

*I always put my patient's satisfaction first and, therefore, provide the best care that I can. If my patient is poor or illiterate, this does not change my behavior toward him or her. I always try to provide quality care for all of my patients.* (P14)

In addition, one participant mentioned the observance of safety in providing care as another dimension of providing quality care, stating:

*You should be very accurate in all of these concerns and duties. You should not forget your patient's medication or mistakenly give their medication twice*. (P1)

#### Effective care

The participants reported that they try to provide effective care in ways that reduce patient pain and improve their condition to achieve desirable caring outcomes. In this regard, the participants mentioned:

*On days that I am working, I will do my best to ensure that my patient feels less pain, so that my care is effective for them. I help my patients, even those who are about to pass away, so that they will pass more comfortably and in less pain.* (P9)

Helping patients be discharged sooner was another standard that the participants considered in providing effective care. One stated:

*The most important thing that I can do is to provide high quality care so that my patient can be discharged sooner and go back to his/her work and life*. (P3)

In addition, the participants mentioned that they would attend to the mental condition of their patients during care and that they would try to reduce patients' concerns and raise their spirits. One of the participants said:

*I do as much as I can for my patient to reduce their concerns. It is important for me as a nurse to be able to relieve my patients' concerns*. (P5)

The lived experiences and remarks of the participants in this regard indicated that the morale of nurses may be improved by the recovery of their patients. One of the participants mentioned:

*Not adding to their problems is the best help for the patient. This also has a positive effect on the nurse's spirit too*. (P4)

#### Individualized care

Avoiding routine-based work and paying attention to the personal differences of patients were some of the measures used by the participants. In this regard, participants mentioned:

*Each patient is different from the others in my eyes. It never becomes repetitious for me. I mean, I do not believe that caring is just physical work. One patient is interested in reading, so I suggest to his/her companions to bring some books or magazines. Another one likes music, so I play music.* (P9)

The experiences of participants in this subcategory indicated that, by paying attention to the needs of their patients, they were working to avoid providing only routine care:

*One thing that I consider really important is evaluating the needs of patients. I mean, the patient's needs must be the basis for our work. The routines are not applicable for everyone*. (P3)

## Discussion

This study described the self-perceived caring motivations of Iranian nurses. One motivation was promoting the exterior effects of the nursing profession by showing the importance of their work to others and creating a positive image in the minds of the patients. Studies have shown that the public image of nurses does not always match their professional image (ten Hoeve, Jansen, & Roodbol, 2014). In Iran, many people think of nurses merely as physicians' assistants (Nasrabadi & Emami, 2006) and the public image of nurses is weak (Alilu, Valizadeh, Zamanzadeh, Habibzadeh, & Gillespie, 2016). According to participants in Iran in the study by Nasrabadi and Emami, nurses are the healthcare workers who are least respected by the general public. A contributing factor for this is the hierarchical relationship between doctors and nurses. This originates from differences in education and from the historical role of nurses and physicians (Nasrabadi & Emami, 2006). The negative and incorrect attitude of people toward nurses is considered as one of the important factors that decreases the motivation of nurses to provide clinical care (Alilu et al., 2016). To modify the reputation of the nursing profession, it is necessary to replace the traditional viewpoint with a more realistic and correct viewpoint. In highlighting the abilities of nurses, the nursing profession is trying to adjust the social position of the nursing profession.

Participants in this study mentioned that professional self-improvement was another concept that motivated them to provide care. Concepts such as self-improvement are in accordance with nursing goals, which include providing care and maintaining internal motivation. Empowerment in the workplace could allow staffs to explore their creativity and provide appropriate solutions in situations that may even lie outside their area of responsibility (Papathanasiou et al., 2014). In Atefi, Abdullah, and Wong (2016), Malaysian nurses mentioned that professional development was one of the most important factors affecting motivation and was critical in improving their knowledge and skills to handle complicated patient needs. These results are all in line with the results of this study, which identified that nurses updating their information by participating in workshops and constant learning was necessary to maintaining motivation to provide care.

According to the results of this study, improving communication skills through professional interactions and providing care was a factor affecting care motivation. Nursing is a combination of knowledge, clinical practice, and interpersonal communications, and as a healthcare science, its performance requires not only scientific knowledge but also interpersonal skills and abilities (Kourkouta & Papathanasiou, 2014). Zhu, Chen, Shi, Liang, and Liu (2016) emphasized that, in addition to revealing the professional values of nurses, this competency should lead to improved nursing care quality and improved nurse–patient relations.

As mentioned earlier, the participants applied value-based behaviors that were beyond their role requirements and were not acknowledged or rewarded directly or publicly by the organization. Studies have revealed that these behaviors not only provide the basis for positive acts and participation but also prepare the ground for tolerating irregular costs, discomforts, and minor frustrations, which are frequently associated with organizational jobs (Lin & Hsiao, 2014) and are considered one of the most important factors affecting the quality of service provision (Hadjali & Salimi, 2012). Furthermore, Osman, Othman, Rana, Solaiman, and Lal (2015) showed that work motivation is strongly associated with these behaviors.

The willingness of nurses to provide beneficial care was a significant finding of this study. In talking about their overall experiences and perceptions of caring motivations, the participants described their work as humane. Helping others, they felt, was based on a humanistic perspective and a desire to help care for sick people. Newton, Kelly, Kremser, Jolly, and Billett (2009) identified four core motivations for individuals to choose a career in nursing: (a) a desire to help, (b) caring, (c) sense of achievement, and (d) self-validation. Newton et al.'s descriptions of the first and second themes are consistent with the findings of this study, in which caring was the dominant theme identified. Nurse participants in the study of Isfahan, Hosseini, Khoshknab, Peyrovi, and Khanke (2015) stated that a deep understanding of patients' clinical conditions and realizing the problems of patients and their families facilitated the emergence of ideas and new solutions that effectively reduced patient/patient–family problems and helped both the patients and hospital colleagues. Nurses have stated that they cannot be indifferent toward the pains and the physical, mental, and financial problems of hospitalized patients and that they would be willing to provide more and better services to the patients (Shahsavari Isfahani, Hosseini, Fallahi Khoshknab, Peyrovi, & Khanke, 2015). Results of this study found that the participants intended to provide safe, standardized nursing care so that they would be able to provide a good quality of care. In Kieft, de Brouwer, Francke, and Delnoij (2014), participants considered technical skills to be an aspect of nursing expertise and critical to providing care that is both effective and safe.

In most previous studies addressing level of motivation in individuals, working conditions were shown to relate positively to both quality of care and the enforcement of safety standards for patients. This indicates the need to incorporate motivation promotion strategies into quality improvement programs at healthcare centers (Alhassan et al., 2013).

### Conclusions

In light of the key role played by nurses in improving patient outcomes, the healthcare system should encourage nurses to think and act beyond their usual roles and to engage in constant learning to maximize healthcare system outcomes in terms of both care outcomes and patient-perceived care quality. The findings and recommendations of this study may be used by senior nursing managers in programs that are designed to improve the quality of nursing care.

### Limitations

The data for this study were gathered from only one healthcare facility and university and thus may not be representative of nurses in other organizations. Although the sample size was substantial and was sufficient in terms of qualitative research standards, the results may not be widely generalizable beyond the study population and sufficiently analogous settings.
